# Stimulating fermentation by the prolonged acceleration of gut transit protects against decompression sickness

**DOI:** 10.1038/s41598-018-28510-x

**Published:** 2018-07-04

**Authors:** Sébastien de Maistre, Nicolas Vallée, Sandrine Gaillard, Claude Duchamp, Jean-Eric Blatteau

**Affiliations:** 1 Service de Médecine Hyperbare et Expertise Plongée, HIA Sainte-Anne, BP600 Toulon, Cedex 9 France; 2 Équipe Résidente de Recherche Subaquatique Opérationnelle, Institut de Recherche Biomédicale des Armées, BP 600 Toulon, Cedex 9 France; 3 Biotech Services, Université de Toulon, CS 60584 Toulon, Cedex 9 France; 4 LEHNA, UMR 5023-CNRS/UCBL, Université Claude Bernard Lyon 1, 43 Bd du 11 Novembre, 1918 Villeurbanne, Cedex France

## Abstract

Massive bubble formation after diving can lead to decompression sickness (DCS). Gut fermentation at the time of a dive exacerbates DCS due to endogenous hydrogen production. We sought to investigate whether medium-term stimulation of fermentation as a result of polyethylene glycol (PEG)-induced acceleration of bowel transit before diving exacerbates DCS in rats. Seven days before an experimental dry dive, 60 rats were randomly divided in two groups: an experimental group treated with PEG (n = 30) and an untreated control group (n = 30). Exhaled hydrogen was measured before the dive. Following hyperbaric exposure, we assessed for signs of DCS. After anaesthetisation, arterial blood was drawn to assay inflammatory cytokines and markers of oxidative stress. PEG led to a significant increase in exhaled H_2_ (35 ppm [10–73] compared with control 7 ppm [2–15]; p = 0.001). The probability of death was reduced in PEG-treated rats (PEG: 17% [95% CI 4–41] vs control: 50% [95% CI 26–74]; p = 0.034). In addition, inflammatory markers were reduced, and the antioxidant activity of glutathione peroxidase was significantly increased (529.2 U.l^−1^ [485.4–569.0] versus 366.4 U.l^−1^ [317.6–414.8]; p = 0.004). Thus, gut fermentation might have a positive effect on DCS. The antioxidant and neuroprotective properties of the fermentation by-products H_2_ and butyrate may explain these results.

## Introduction

Given that the vast majority of decompression sickness (DCS) events occur although diving procedures have been followed by divers, we seek to verify whether intrinsic factors, such as gut fermentation (present before the dive), may be involved in DCS risk.

Diving is a risky activity that can lead to bubble formation in the bloodstream and tissues as a result of dissolved gas changing phases during an ascent. The level of venous gas emboli is associated with the risk of DCS^1^. When too many bubbles are generated, signs and symptoms of DCS can occur^2^. It is generally accepted that these bubbles form from pre-existing gaseous nuclei attached to vessel walls^3^. Bubbles cause cell damage^4^, prothrombotic phenomena, ischaemia and diapedesis^5,6^. The inflammation can spread systemically and may degenerate into a vicious cycle. Spinal cord and brain neurological damage underlie the most serious and common symptoms of DCS. Even after standard treatment with hyperbaric oxygen, 20–30% of victims suffer sequelae after neurological DCS^7^. Identifying and managing factors that might influence the risk of DCS is therefore an important issue.

It has been shown that the gut microbiota can affect the risk of DCS. In rats and humans diving with air, bacterial fermentation of undigested sugars, which is associated with an increase in hydrogen production at the time of diving, is accompanied by an increase in the incidence of DCS^8,9^. Similarly, it has been demonstrated that increased removal of diluent hydrogen by gut microbes can affect the risk of DCS, and both native and supplemental addition of hydrogen-metabolizing microbes help reduce DCS risk^10–14^. Given that hydrogen can diffuse across the gut wall, disperse throughout the body and contribute to the initial formation of inert gas bubbles from nuclei, i.e., the micro-bubbles that serve as the origin of desaturation events^3^, we also suggest a fraction of the H_2_ generated by excessive fermentation in the gut could have a harmful effect during decompression by directly increasing the amount of inert gas. It has been widely demonstrated that bowel transit time affects the gut microbiota and fermentation. In both humans and animals, rapid bowel transit is associated with increased faecal mass and an increased concentration of H_2_ in exhaled air, which reflects increased fermentation^15–17^.

Bowel transit time is reduced in some conditions. In patients with short bowel syndrome, oro-caecal transit is accelerated, and fermentation capacity is increased^18^. Stress is also accompanied by accelerated transit throughout the digestive tract^19^. Bowel transit can also be accelerated in normal circumstances, e.g., as a result of aerobic physical exercise^19^ or following the consumption of unfermentable cellulose^20^. Bowel transit may also be accelerated pharmacologically. High-molecular weight polyethylene glycol (PEG) is a long linear polymer of ethylene glycol monomers that binds water molecules through hydrogen bonds. PEG cannot be digested or fermented. When administered orally, PEG increases the volume of fluid present in the gut, explaining the solution’s underlying laxative activity. PEG increases bowel transit in both the small intestine^21–23^ and colon^24^. In non-fasting mice inoculated with human faecal micro-organisms, long-term PEG administration increases transit, induces changes in the composition of the gut microbiota similar to those observed on a diet based on unfermentable cellulose, and increases fermentation capacity^20^.

The aim of these experiments was to investigate whether medium-term stimulation of fermentation by accelerating bowel transit increases the risk of DCS. Before the dive, rats were fed PEG for a week to stimulate gut fermentation. We initially decided to assess the effect of PEG ingestion at the clinical level on rats more likely to report DCS, i.e., on heavier rats (H), following pathogenic decompression. Then, the impact of this treatment was assessed on selected rats less likely to report DCS (low-risk population or lighter rat: L), in which blood cells counts and biochemical analyses could be performed more accurately, i.e., without interference from the major clinical symptoms of DCS (such as death).

## Results

### Clinical study in the at-risk population

#### Population characteristic: Body weight/fluid taken in

On the day of pressurisation, no difference in body mass was noted between the 36 polyethylene (PEG) and control (CTRL) rats (PEG_H_ vs CTRL_H_: n = 18/18, 409 g [403–421] versus 392 g [385–416]; p = 0.205). The volume of fluid taken in was not different between the two groups (PEG_H_ vs CTRL_H_: 295 ml [270–340] vs 289 ml [256–327], p = 0.438).

#### Gut activity and fermentation activity: Stools/Caecum with Short-chain fatty acids/Hydrogen and carbon dioxide in exhaled air

**Figure 1:**
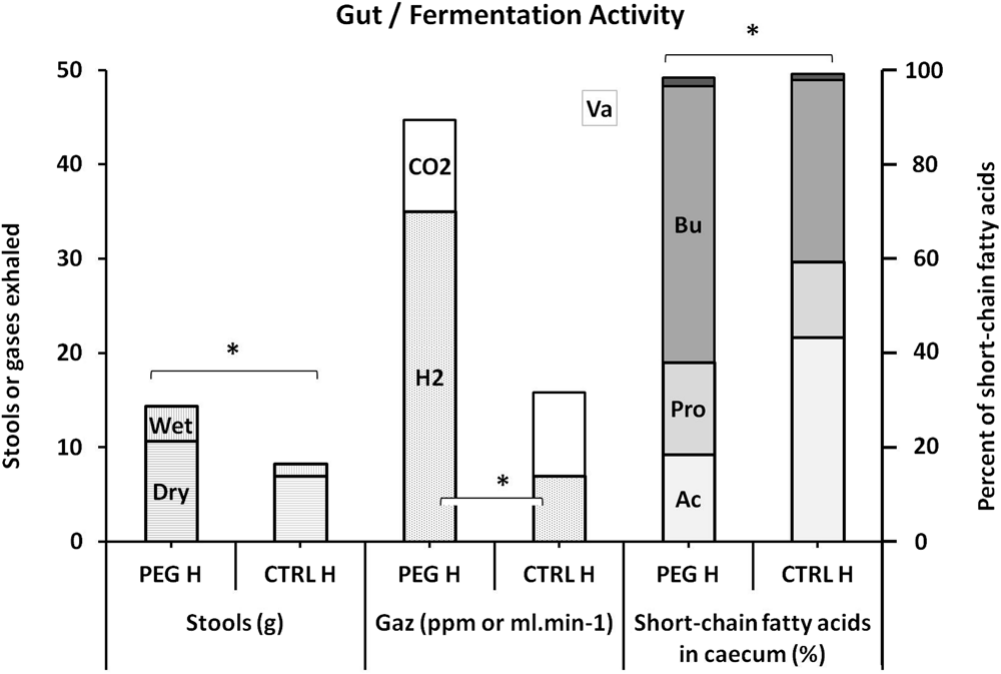
Gut and fermentation activities in the at-risk population (Bu: butyrate, Pro: propionate, Ac: acetate, Va: valerate). *Denotes p < 0.05 between the groups.

The weight of stools collected over two days was significantly increased in the PEG group (PEG_H_ vs CTRL_H_: 14.3 g [12.8–16.3] vs 8.3 g [8.0–9.0], p = 0.0001). The same was true for the weight of water in stools (PEG_H_ vs CTRL_H_: 3.7 g [2.6–8.6] vs 1.3 g [1.1–2.0], p < 0.0001) and their dry weight (PEG_H_ vs CTRL_H_: 10.7 g [9.7–11.9] vs 7.0 g [6.2–7.2], p = 0.0001) (Fig. 1).

Caecum weight was also significantly increased in the PEG group (PEG_H_ vs CTRL_H_: 11.5 g [10.6–13.2] vs 6.9 ml [6.3–8.0], p < 0.0001).

The proportion of butyrate in the caecal content was significantly increased in the PEG group (PEG_H_ vs CTRL_H_: 58.4% [53.0–65.4] vs 38.5% [32.4–40.7], p < 0.0001). The same finding applies to a lesser extent for propionate (PEG_H_ vs CTRL_H_: 19.6% [17.0–23.8] vs 16.0% [14.0–20.1], p = 0.052) and valerate (PEG_H_ vs CTRL_H_: 1.9% [1.5–2.8] vs 1.4% [1.2–1.7], p = 0.005). On the other hand, the proportion of acetate in the caecal content was significantly reduced in the PEG group (PEG_H_ vs CTRL_H_: 18.5% [10.0–23.4] vs 43.3% [40.8–47.4], p < 0.0001) (Fig. 1). A Spearman test detected a positive correlation between the proportion of butyrate in the caecal content and the weight of stools collected over two days (PEG_H_ and CTRL_H_ Spearman_Butyrate/stools:_ n = 36, α = 0.05, p = 0.0002).

One hour before pressurisation, significantly more H_2_ was noted in the air exhaled by PEG_H_ group rats (35 ppm [7–77] compared with 7 ppm [4–15], p = 0.047) (Fig. 1). A Spearman test detected a positive correlation between H_2_ in exhaled air and the weight of stools collected over two days (PEG_H_ and CTRL_H_: Spearman_H2/stools:_ n = 36, α = 0.05, p = 0.049). In contrast, no significant difference was noted between the levels of CO_2_ produced one hour before pressurisation by the PEG_H_ and CTRL_H_ groups (PEG_H_ vs CTRL_H_: 9.7 ml.min^−1^ [8.2–12.3] vs 8.8 ml.min^−1^ [7.6–12.6], p = 0.281).

#### Blood cell counts

Before the dive, no differences in blood cells were noted between groups, confirming the lack of a treatment effect on the blood components (leukocytes, erythrocytes, and platelet count). In the PEG_H_ and CTRL_H_ groups, no significant differences in the haematocrit four hours before pressurisation (56.4% [52.6–59.8] compared with 54.0% [48.2–59.0]; p = 0.235) or after (p = 0.812) were observed between the two groups, suggesting a similar hydration status.

#### Clinical effect of the provocative dive in the at-risk population

The dive protocol resulted in DCS cases, especially in at-risk groups (PEG_H_ and CTRL_H_), and the general symptoms included alterations in physical and behavioural performances, including decreased scores in all locomotor and neurological tests, among all groups included.

**Figure 2:**
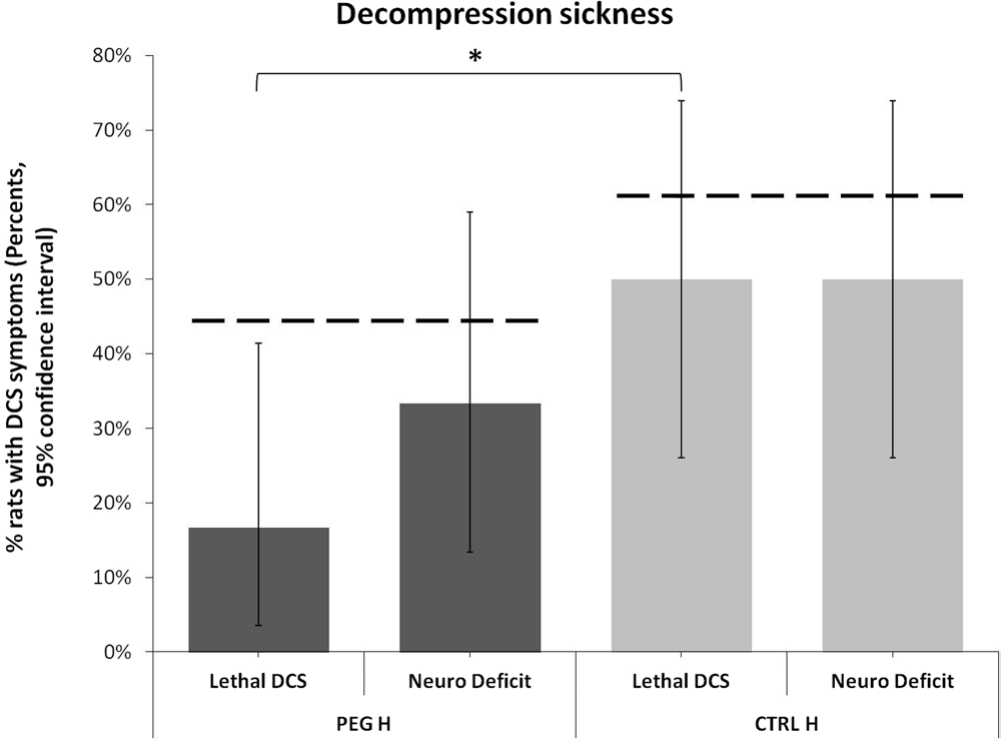
Decompression sickness within 30 min after surfacing in the at-risk population. *Denotes p < 0.05 between the groups.

Although no significant differences in the proportion of DCS were observed in the two at-risk groups, differences in severity were noted between the groups with notably increased mortality observed in untreated rats (Fig. 2). One convulsion was also noted in the CTRL_H_ group. Significantly fewer deaths were noted in the PEG_H_ group (17% [95%CI 4–41] versus CTRL_H_ rats 50% [95%CI 26–74], p = 0.034), and this group scored better in the righting reflex (2.0 [2.0–2.0] compared with 1.4 [0.3–2.0]; p = 0.014). Animals in this groups also tended to score better on the SFI test (2.0 [2.0–2.0] compared with 1.4 [0.0–2.0]; p = 0.097).

In the at-risk population, the weight of stools collected over two days was significantly increased in those that survived compared with those that died (13.00 g [9.09–14.91] compared with 8.38 g [8.08–9.31]; p = 0.0056) regardless of PEG treatment. In addition, slightly more H_2_ was noted in the air the survivor rats were exhaling one hour before pressurisation (22 ppm [6–59] compared with 7 ppm [3–16]; p = 0.0777). A Spearman test detected a positive correlation between the beam walk score and the weight of stools collected over two days (PEG_H_ & CTRL_H_, Spearman_beam/stools:_ n = 36, α = 0.05, p = 0.009).

### Clinical and biological study in the low-risk population

#### Population characteristic: Body weight/Fluid taken in

On the day of pressurisation, there was no difference in weight between the 24 polyethylene and control rats (PEG_L_ and CTRL_L_: 368 g [361–371] versus 370 g [360–379]; p = 0.544).

The volume of fluid taken in was not different between groups (PEG_L_ vs CTRL_L_: 313 ml [289–359] vs 327 ml [243–364], p = 0.997).

#### Gut activity and fermentation activity: Stools/Caecum with Short-chain fatty acids/Hydrogen and carbon dioxide in exhaled air

**Figure 3:**
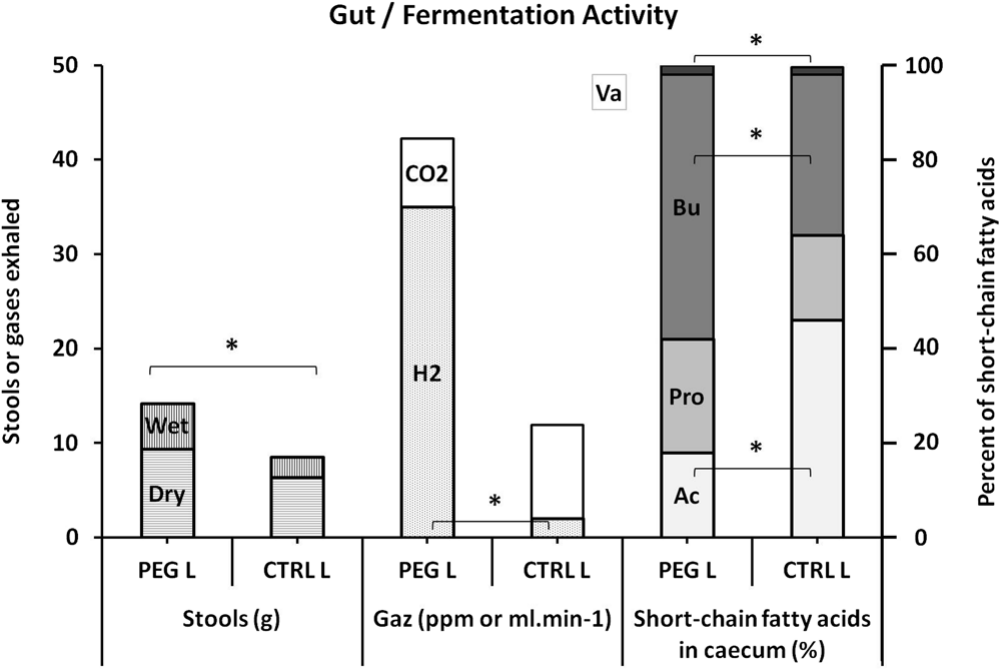
Gut and fermentation activities in the low-risk population (Bu: butyrate, Pro: propionate, Ac: acetate, Va: valerate). *Denotes p < 0.05 between the groups.

The weight of stools collected over two days was significantly increased in the PEG group (PEG_L_ vs CTRL_L_: 15.4 g [11.6–17.7] vs 8.7 g [6.6–11.3], p = 0.005). The same was true of the weight of water (PEG_L_ vs CTRL_L_: 4.8 g [4.4–6.9] vs 2.1 g [1.8–2.5], p = 0.003) and dry weight (PEG_L_ vs CTRL_L_: 9.4 g [7.1–11.2] vs 6.4 g [4.9–8.1], p = 0.035) (Fig. 3).

Caecum weight was also significantly increased in the PEG group (PEG_L_ and CTRL_L_: 11.7 g [10.3–13.3] vs 7.1 g [4.6–7.8], p < 0.0001).

The proportion of butyrate in the caecal content was significantly increased in the PEG group (PEG_L_ vs CTRL_L_: 56.8% [39.1–65.5] vs 34.8% [30.1–35.8], p = 0.045). The same finding applies for valerate (2.2% [1.9–2.4] vs 1.6% [1.2–1.9], p = 0.018) but not for propionate (PEG_L_ and CTRL_L_: 25.1% [18.9–26.3] vs 18.6% [15.4–24.6], p = 0.207). On the other hand, the proportion of acetate in the caecal content was significantly reduced in PEG group (PEG_L_ and CTRL_L_: 18.9% [8.2–40.7] vs 43.7% [40.1–45.9], p = 0.029) (Fig. 3). The Spearman test failed to detect a positive correlation between the proportion of butyrate in the caecal content and the weight of stools collected over two days (Spearman_Butyrate/stools:_ n = 23, α = 0.05, p = 0.111).

One hour before pressurisation, significantly more H_2_ was noted in the air exhaled by the PEG group rats (PEG_L_ vs CTRL_L_: 35 ppm [21_50] vs 2 ppm [1_19], p = 0.011). A Spearman test detected a positive correlation between H_2_ in exhaled air and the weight of stools collected over two days (Spearman_H2/stools:_ n = 23, α = 0.05, p = 0.028). In contrast, no additional CO_2_ was produced one hour before pressurisation in the PEG group (PEG_L_ and CTRL_L_: 7.2 ml.min^−1^ [6.7–9.1] vs 9.9 ml.min^−1^ [7.1–12.0], p = 0.147).

#### Clinical effect of the provocative dive in the low-risk population

As expected for the PEG_L_ and CTRL_L_ rats, very few symptoms of DCS were observed. Only one PEG_L_ rat died, and one rat from each group (PEG_L_ & CTRL_L_) exhibited objective signs of neurological damage. Indeed, no significant difference was noted between those groups.

#### Blood analysis

Blood cell counts: Before the dive, no difference in blood cells was noted between PEG_L_ and CTRL_L_ groups, confirming that the treatment had no effect on blood cell counts (leukocytes, erythrocytes, and platelet count). In the PEG_L_ and CTRL_L_ groups, no significant differences in the haematocrit four hours before pressurisation (46.6% [44.8–48.6] compared with 48.0% [45.8–51.4]; p = 0.441) or after (p = 0.393) were observed between the two groups.

**Figure 4:**
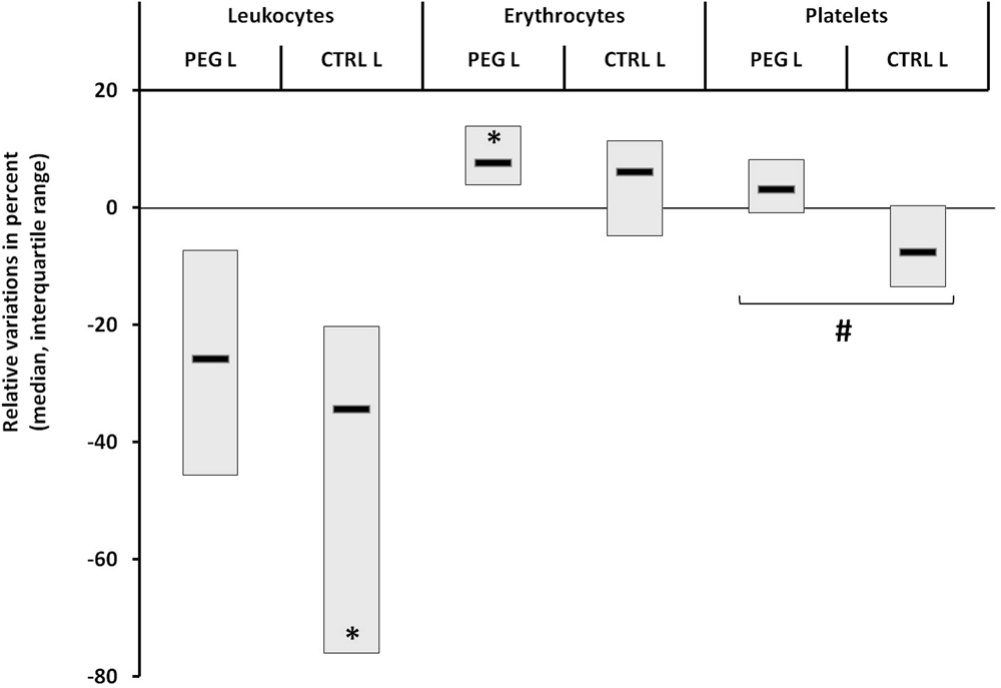
Relative variations of blood cell counts after hyperbaric exposure in the low-risk population. *Denotes p < 0.05 between paired groups and #denotes p = 0.066 between unpaired groups.

Following the dive, a significant decrease in the leukocyte count after hyperbaric exposure was noted in the CTRL_L_ group (−34.3% [−76.0 to −20.3], W p = 0.011) but not in the PEG_L_ group (−25.9% [−45.7 to −7.3], W p = 0.266). A small increase in the erythrocyte count was noted upon removal from the hyperbaric chamber in the PEG_L_ group (7.6% [3.8–13.9], W p = 0.045) but not in the CTRL_L_ group (6.1% [−4.8–11.4], W p = 0.405). Finally, the variation in the platelet count tended to differ between these two groups (PEG _L_ 3.1% [−0.9–8.1] vs CTRL _L_ −7.7% [−13.5–0.3], MW p = 0.066). Taken together, these results allow us to hypothesize that CTRL_L_ rats could have been slightly more affected by the provocative dive (Fig. 4).

#### Inflammatory cytokines and markers of oxidative stress in the blood

**Figure 5:**
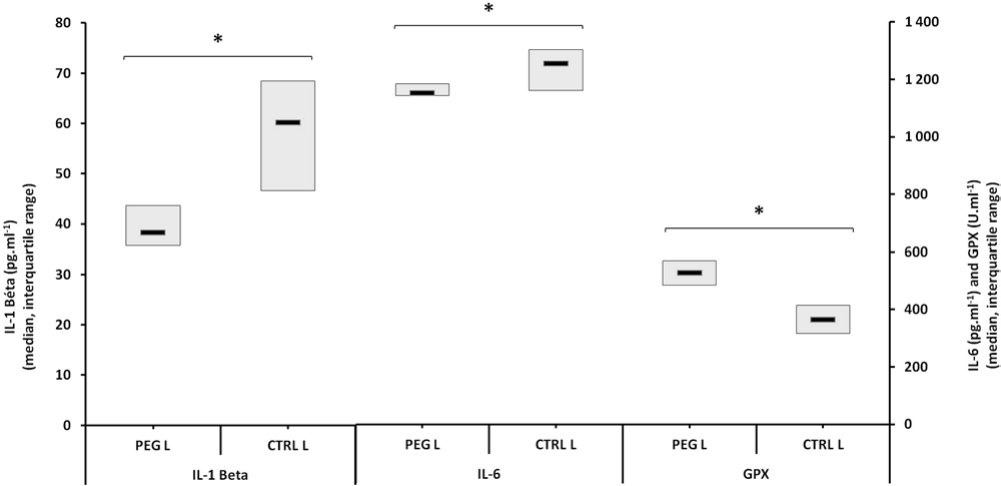
Pro inflammatory cytokines and markers of oxidative stress in the blood of the low-risk population. *Denotes p < 0,05 between the groups.

In the PEG_L_ and CTRL_L_ groups, the levels of inflammatory cytokines in arterial blood after anaesthesia were significantly reduced in the PEG group: IL-1β (38.3 pg.ml^−1^ [35.7_43.6] versus 60.2 pg.ml^−1^ [46.6_68.4], p = 0.001) and IL-6 (1153.6 pg.ml^−1^ [1144.5–1184.5] versus 1257.1 pg.ml^−1^ [1162.6–1303.7], p = 0.049) (Fig. 5).

Moreover, glutathione peroxidase activity was significantly increased in the PEG group (529.2 U.l^−1^ [485.4–569.0] versus 366.4 U.l^−1^ [317.6–414.8], p = 0.004) (Fig. 5). A Spearman test revealed a tendency towards a positive correlation between glutathione peroxidase activity and the amount of H_2_ in the exhaled air (PEG_L_ and CTRL_L_ group, Spearman_GPX/H2_, p = 0.087). No significant difference emerged with respect to TBARS (0.66 µmol.l^−1^ [0.54–0.75] malondialdehyde (MDA) equivalents versus 0.57 µmol.l^−1^ [0.45–0.59], p = 0.495).

## Discussion

Although previous works seem to suggest that the risk of DCS increases according to hydrogen production (i.e., fermentation), our results highlight the fact that the risk decreases when fermentation is stimulated for one week.

### PEG-induced fermentation

In rats fed a standard diet, we demonstrated that the ingestion of PEG for 7 days before a dive increases faecal production concomitant with enhanced hydrogen exhalation, and the latter of which reflects gut fermentation. Actually, this increase in faeces is accompanied by an increase in its dry and water content. Given that the haematocrit is unchanged, we can dismiss the possibility of dehydration. As a result of fermentation, the composition of faeces varies from one group to another with the treated rats having proportionally more butyrate, propionate or valerate and less acetate than the other groups. The increase in butyrate was at the expense of the acetate, as is described in fermentation processes occurring in the intestine^25^. Finally, the increased faecal mass and hydrogen exhaled in air recorded in this study suggest rapid bowel transit and increased rate of fermentation, as suggested by various authors in both rats^17^ and humans^15,16^.

### Decompression sickness

DCS cases were generated using the same dive protocol as described in previous studies^4,9,26^. Globally, DCS manifests as an alteration in physical and behavioural performances accompanied by a deterioration in biological constants.

In contrast to what was expected, this enhanced fermentation resulted in a significant reduction in the incidence of DCS based on the following mechanisms: (i) reduced mortality and sciatic nerve dysfunction in the at-risk population and (ii) reduced levels of circulating interleukins and blood parameters alterations (suggesting less premises for DCS) in the low-risk population. In control rats, in particular, we measured significant decreases in the platelet count mostly attributed to the interaction between bubbles and platelets in DCS^27^ as well as the leukocyte count. This finding is also consistent with the increase in pro-inflammatory IL-1 beta, suggesting that inflammation causes diapedesis^26,27^.

### Medium-term fermentation cuts down the risk of DCS

Taken together, these results suggest medium-term gut fermentation reduces the risk of DCS in rats. Thus, it seems that faster bowel transit could protect against DCS.

#### Fermentation effect on gas load and bubble-induced DCS

As stated above, increasing the water content of faeces potentially modified the hydration level and therefore increased the DCS risk, but this modification does not change the haematocrit or increase DCS symptoms in PEG-treated rats.

At first glance, the findings reported here might seem to contradict our previous results^9^. In both studies, the aim was to stimulate gut fermentation and H_2_ production.

In the course of hyperbaric exposure, the surplus load of inert gas in the form of H_2_ generated by bacterial fermentation was assumed to induce a significant increase in bubble volume during decompression and promote DCS. In fact, this process did not occur in this study. It seems that insufficient H_2_ was generated inside the body to exacerbate the risk of DCS in rats. In fact, the levels of H_2_ measured in exhaled air one hour before these dives were significantly reduced compared with those reported previously (35 ppm [10–73] compared with 119 ppm [76–166]; p = 0.0003)^9^. In addition, although we previously stimulated H_2_ production by administering a bolus dose of the fermentable mannitol four hours before the dive^9^, we only undertook a medium-term acceleration of bowel transit for a full 7 days before the dive and without a highly fermentable substance in this study.

This finding suggests that protection from DCS by medium-term gut fermentation may require some time to benefit from the neuroprotective effects of endogenous H_2_ and butyrate, potentially explaining why no such effect was observed in the previous experiments^9^.

#### Hydrogen and butyrate could protect from DCS

Protective activity of H_2_: We observed a tendency towards a positive correlation between exhaled H_2_ and the activity of glutathione peroxidase, an enzyme with antioxidant properties. We suggest here that fermentation provides protection from DCS by the virtue of an antioxidative system. This hypothesis relies on the over-production of ROS (reactive oxygen species) and HSP (heat shock protein) in a dive^28^. It was also recently demonstrated in humans that successive deep dives have a negative effect on endothelial function and induce oxidative stress^29^. In a previous study, we demonstrated that endogenous H_2_ production as a result of bacterial fermentation could help prevent DCS^9^. Intraperitoneal administration of H_2_-enriched normal saline over the 24-hour period before a high-risk dive can prevent DCS in rats^30^. H_2_ can act as an antioxidant by selectively reducing the hydroxyl radical (OH^•^) and peroxynitrite anion (ONOO^−^); however, H_2_ does not react with other biologically active reactive oxygen intermediates^31^. The same group demonstrated that inhaling H_2_ gas attenuates brain damage following focal ischaemia/reperfusion injury in rats by neutralizing the effects of oxidative stress. The mechanism of action underlying this phenomenon involves both anti-inflammatory and anti-apoptotic effects^32–34^. This result parallels results on cytokines and leukocytes presented in this study. H_2_ also limits broaching of the blood-brain barrier by reducing reactive oxygen intermediates and inhibiting the activity of matrix metalloproteinase-9^35^. Other studies have demonstrated that inhaled H_2_ can have a positive effect on medullary ischaemia-reperfusion lesions in rabbits^36^. Finally, endogenous H_2_ produced by fermentation after the ingestion of lactulose exerts neuroprotective effects with respect to ischaemia-reperfusion lesions in the rat brain^37^; the same mechanism could apply to DCS.

Butyrate: Gut gases do not exclusively include metabolites generated by bacterial fermentation of indigestible carbohydrates. Quantitative analyses of products of *in vitro* fermentation of lactulose have demonstrated that the main non-gaseous metabolites include acetic, lactic and butyric acids, which are all typically produced by *Clostridium* spp^38^. In addition, butyrate, a short-chain fatty acid that is increased in PEG rats in this study, is known for its neuroprotective activity. As an inhibitor of histone deacetylase, butyrate has antioxidant^39^, anti-inflammatory and anti-apoptotic effects^39–41^. The activation of microglial cells and macrophages/monocytes induced by definitive ablation of the middle cerebral artery, which damages white matter, is inhibited by butyrate^40^. Moreover, an association can be made with lower levels of variation in cytokines and leukocyte counts after the dive of the treated rats of this study. In these cases with the neurological form of DCS _SFI being reduced in PEG rats, it is of interest to note that hydroxybutyrate also significantly reduces brain damage and more globally cell death^39,42^.

#### A biphasic effect

**Figure 6:**
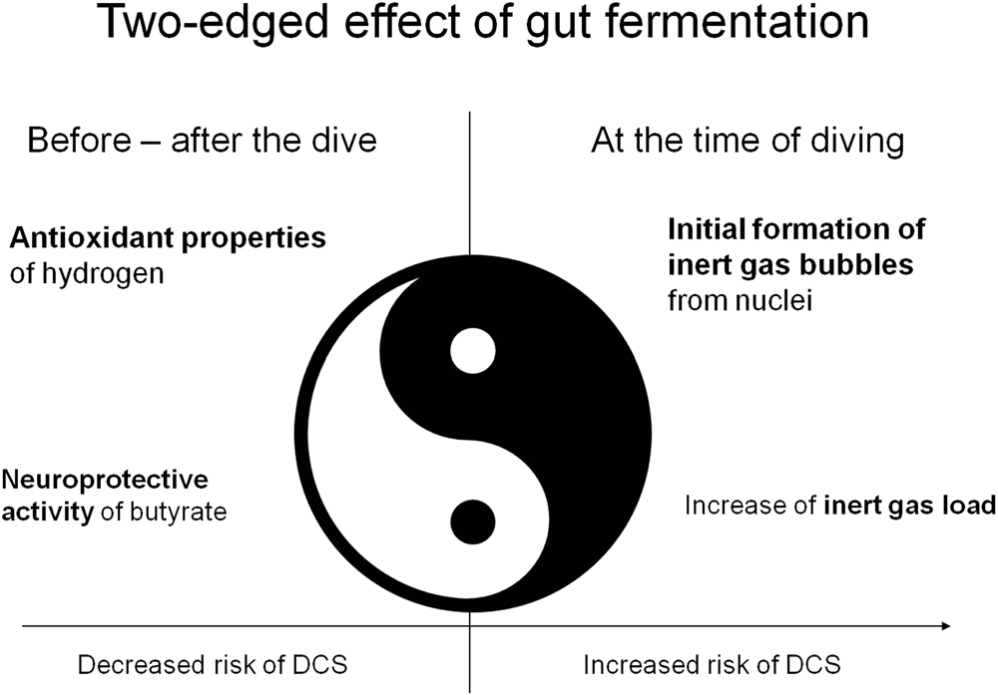
Summary of the effects of intestinal fermentation on the risk of decompression sickness.

Our findings point to a two-edged effect of gut fermentation on decompression. Despite being detrimental in the short term, i.e., at the time of diving, gut fermentation apart from dives might have a positive effect by preventing the occurrence of DCS and limiting its severity (Fig. 6). Thus, it might seem wise to reduce consumption of foodstuffs with high fermentation potential the day before a dive. In addition, any factor that might affect the gut microbiota and stimulate fermentation (prebiotic and probiotic products) could be tested to investigate its potential in protecting against DCS.

### Could it be applied in human dives?

#### To prevent DCS

An association between modification of the gut microbiota and increased fermentation was previously demonstrated in a number of cases, i.e., using prokinetic agents, such as cisapride in humans^15^ and bisacodyl in rats^17^. Regarding other molecules, an increase in the fermentation rate is expected from the inversely proportional relationship between bowel transit time and the amount of H_2_ in exhaled air^15–17^. This situation occurs with certain antiemetic agents, such as metoclopramide and domperidone, which affect oro-caecal transit time^43^. Regular physical exercise can also stimulate fermentation by accelerating oro-caecal transit^19^ and experiments on humanized mice (i.e., germ-free mice inoculated with human faecal microbiota) have demonstrated that a diet based on unfermentable cellulose increases gastrointestinal transit and induces changes in the gut microbiota similar to those observed after the administration of PEG^20^. In turn, short-chain fatty acids, which result from gut fermentation, could also stimulate fermentation by increasing the rate of transit through the small intestine^44^. H_2_ produced in the colon could accelerate transit in this portion of the gut, especially the proximal colon^45,46^.

#### To treat DCS

Due to lack of control of the gastrointestinal system by the central nervous system, many people with spinal cord damage suffer from impaired gut function with effects on bowel transit time. Specifically, the density of butyrate-producing bacteria is reduced in individuals with spinal cord damage compared with healthy subjects^47^. Moreover, butyrate seems to have the ability to repair the blood-brain barrier by stimulating the expression of proteins found in tight junctions^48^. By inhibiting histone deacetylase, butyrate also stimulates neurogenesis and could be implicated in the later stages of neurovascular recovery^40^. Cell proliferation, migration and differentiation induced by the inhibition of histone deacetylase involve the brain-derived neurotrophic factor and its receptor tropomyosin receptor kinase B^40^. Butyrate is a potent stimulant for vascular endothelial growth factor, which plays central roles in neurogenesis, angiogenesis and functional recovery after ischaemic stroke^40^. Thus, replenishing butyrate-producing bacteria could also be effective in the management of neurological DCS.

Given the lack of knowledge, further experiments will be required to investigate the various factors that can accelerate bowel transit with the aim of preventing or treating DCS.

## Conclusion

Our findings suggest that in contrast to what was expected medium-term stimulation of fermentation seems to reduce the risk of DCS in rats. Our findings point to a two-edged effect of gut fermentation on decompression. Although detrimental in the short term, i.e., at the time of diving, gut fermentation that occurs in the absence of diving might have a positive effect by preventing the occurrence of DCS and limiting its severity. H_2,_ which has antioxidant properties, and butyrate, a short-chain fatty acid, are both by-products of the fermentation of carbohydrate, and both could exhibit interesting neuroprotective activity. Stimulation of fermentation could also play a positive role when in the treatment of DCS.

## Materials and Methods

**Figure 7:**
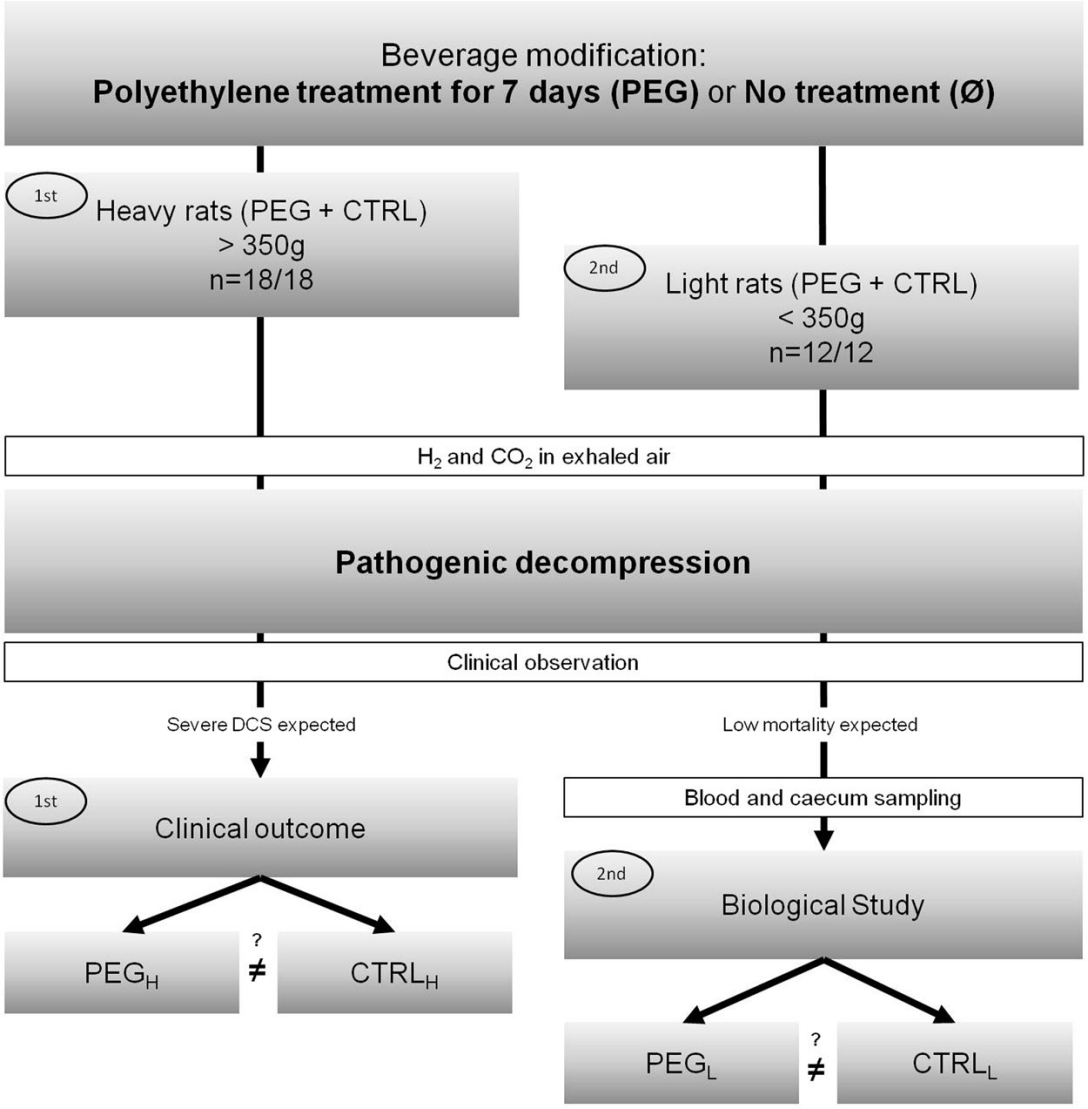
Distribution of rats in experimental groups.

The experimental design is presented in Fig. 7.

### Study Population

All procedures involving experimental animals adhered to European Union rules (Directive 2010/63/EU) and French law (Decree 2013/118). The Ethics Committee of the Institut de Recherche Biomédicale des Armées approved this study (C2EA-SSA). Male Sprague-Dawley rats (Charles River Laboratory, France) were maintained in an accredited room at a temperature of 22 ± 1 °C with a day/night cycle of 12 hours each (light turned on at 7 a.m.). Before the experiments, the rats were fed a standard diet (12 mm granules from Global Diet 2018, Harlan, Italy) and water. Sixty rats were included in this study.

### Drug and Treatment

The experimental group was treated with PEG for seven days before pressurisation. The treated rats were administered water supplemented with Fortrans® (IPSEN Pharma, France) (one sachet per litre, i.e., 64 g PEG 4000 per litre). The water that was administered to the control rats (CTRL) contained the same additives as Fortrans® without PEG. Throughout the treatment phase, the rats were housed in individual cages, and their food intake was controlled to maintain a constant weight (16 g granules a day).

### Fluid consumed and stools produced

The amount of fluid consumed over 7 days was estimated by measuring the volume remaining in the rat’s bottle on the day of pressurisation. Consumption was normalised to each rat’s body weight.

Over the 48 hours before pressurisation, the weight of the faeces produced by each rat was measured. The litter was replaced two days before pressurisation. On the day of pressurisation, the litter was sieved to harvest all the stools passed over the last two days. These stools were then weighed, dried in an oven at 60 °C for 24 hours, and then weighed again to determine their dry weight.

### Hydrogen and carbon dioxide in exhaled air

For all rats, the amount of H_2_ measured in exhaled air provides a measurement of the rate of H_2_ production resulting from the bacterial fermentation of carbohydrates in the gut (and hence diffusing throughout the body via the bloodstream). The amount of CO_2_ in exhaled air depends on both the host’s metabolic activity and bacterial fermentation in the digestive tract (which diffuses across the gut wall).

To measure H_2_ and CO_2_ in exhaled air, we considered that the breathing rate in rats is constant over time with a mean rate of 225 ml.min^−1^. The literature provides a resting value of 27.27 ± 2.39 ml.min^−1^.100 g^−1^ (mean ± standard deviation) for Sprague-Dawley rats^49^. The mean weight of the rats we studied was 389 ± 24 g (mean ± standard deviation). However, taking into account the stress induced by the measurement process, we used a breathing rate double that of the value in the literature for unstressed, resting rats.

For each measurement, each rat was placed in a clean, dry polyvinyl chloride (PVC) cylinder (internal diameter 75 mm, length 200 mm, i.e., an internal volume of 883 ml). Both ends were hermetically sealed with plastic discs with holes in the middle, allowing air in at one end and the collection of gases at the other end. Air was circulated by a special aerator (Rena Air 200®, France) set to a constant flow rate of 225 ml ml.min^−1^ that was controlled by the rise of a soap bubble in an inverted 100-ml test tube that was pierced at the bottom.

After 5 minutes (to allow time for the gases to mix inside the cylinder, taking account of the dead space, i.e., that not occupied by the animal), successive measurements of H_2_ (ppm) and then CO_2_ (percentage) in the air coming out of the cylinder were performed by means of a three-way tap.

H_2_ was measured using a mobile exhaled H_2_ analyser (Gastrolyser®, Respur International, France), and CO_2_ was measured using a mobile gas exchange test system (Cosmed® K4b², Italy). The results were recorded one hour before pressurisation. An illustration of the installation used to measure H_2_ and CO_2_ in exhaled air in rats can be found in a supplementary information file.

### Hyperbaric procedure

Bubble formation and the incidence of DCS are highly dependent on body weight^50–53^. According to our previous study^54^, this protocol using rats with a body weight greater than 350 g produces severe DCS with clear neurological involvement and death. All 60 rats, including 36 heavier rats (H) (weight of over 350 grams) and 24 lighter rats (L) (weight of less than 350 grams), were subjected to hyperbaric exposure followed by decompression to induce bubble formation and DCS. The aim was to obtain clear neurological signs and deaths in the Heavy rats (PEG_H_ & CTRL_H_) and to induce non-fatal manifestations in Light rats (PEG_L_ & CTRL_L_), which should not necessarily be detectable in a physical examination but should induce changes in blood tests.

Batches of 8 rats free to move around and split between two cages (4 per cage) were subjected to a compression/decompression protocol in a 200-litre hyperbaric chamber with three observation ports. Each batch contained both treated (PEG) and control (CTRL) rats.

All rats (PEG_H+L_ & CTRL_H+L_) were subjected to air pressurisation at a rate of 10 kPa.min^−1^ up to 100 kPa followed by a rate of 100 kPa.min^−1^ up to 1.000 kPa (90 msw), which was maintained for 45 minutes. At the end of the exposure period, the rats were decompressed to 200 kPa at a rate of 100 kPa.min^−1^ with a 5-minute stop at 200 kPa, a 5-minute stop at 160 kPa, and a 10-minute stop at 130 kPa. Decompression between 200 kPa and the surface was conducted at a rate of 10 kPa.min^−1^. The decompression rate was controlled automatically by a computer connected to an analogue/digital converter (NIUSB-6211®, National Instrument, USA) that was connected to a solenoid valve (Belino LR24A-SR®, Switzerland) and a pressure sensor (Pressure Transmitter 8314, Burket Fluid Control System, Germany). The software used to regulate the decompression rate was coded by our engineer using DasyLab (DasyLab®, National Instrument, USA).

Compressed air was generated using a diving compressor (Mini Verticus III, Bauer Comp, Germany) connected to a 100-litre buffer bottle at 3.10^4^ kPa. The oxygen analyser was based on a MicroFuel electrochemical cell (G18007 Teledyne Electronic Technologies/Analytical Instruments, USA). Water vapour and CO_2_ produced by the animals were captured with Seccagel (relative humidity: 40–60%) and soda lime (CO_2_ < 300 ppm), respectively. Gases were mixed by an electric fan. The day-night cycle was respected throughout the experiment. The temperature inside the hyperbaric chamber was measured using a platinum resistance temperature probe (Pt 100, Eurotherm, France). All these variables were controlled by a dedicated computer.

### Clinical observation

At the end of decompression, Heavy rats (PEG_H_ & CTRL_H_) were transferred into individual cages and observed at will for 30 minutes by trained observers who were unaware of the treatment. The following were considered as signs of DCS: motor deficit or difficulty moving around (including limping, failure to maintain balance, sideways gait, falling, and difficulty getting up after a fall) and death. Motor deficit was considered as anything below Level 4 on the Gale’s “Motor Score” scale^55^. The time at which any sign was observed was recorded. Possible neurological damage was also assessed at higher resolution by means of a beam walk test that involved seven 110-cm long wooden planks of different widths (7.7–1.7 cm) raised 110 cm above the floor. The rats were first placed on the widest plank, and their ability to cross the plank without slipping was observed twice. This test was repeated with successively smaller planks, and the test was scored as the narrowest plank that the rat could cross, yielding a score of between 0 (dead) and 7^56^.

The toe gap reflex test^57^ evaluates motor function and more specifically sciatic nerve function injury (SFI). The test is based visually on the toe gap where 2 represents a normal state, 1 a small gap and 0 a complete inability to spread the toes.

The righting reflex was elicited by holding the rat in one hand and turning it over on its back 7 or 8 cm above a covered table surface. The way the animal tried to regain its original position with its feet down was studied. To avoid injuring the rats when testing for righting, the animals with a motor score below 3 were tested for their ability to turn by dropping them from a height of 2 or 3 cm. Righting was scored as follows: 0, no attempt to right itself; 1, weak or delayed attempt to right or rights itself in the direction of the roll; 2, normal righting counter to the direction of roll.

### Blood cell counts

Counts were made on an automatic analyser (ABCvet®, SCIL, France) in samples taken four hours before pressurisation and then 30 minutes after removal from the hyperbaric chamber in the low-risk population. Red blood cells, haematocrit, leukocytes and platelets were counted in 20 µl samples taken from the tip of the tail and diluted in an equivalent volume of 2 mmol.l^−1^ EDTA (Sigma, France). The second test values were corrected according to the hematocrit variation.

### Samples taken after anaesthetisation

Thirty minutes after removal from the hyperbaric chamber, the rats were anaesthetised with an intraperitoneal injection of a mixture of 10 mg.kg^−1^ xylazine (Rompum® 2%, Bayer Pharma, Germany), 100 mg.kg^−1^ ketamine (Imalgène®1000, Merial, France) and 1.65 mg.kg^−1^ acepromazine (Calmivet®, Vetoquinol, France).

At the end of the study, the rats were sacrificed by means of an intraperitoneal injection of pentobarbital (200 mg.kg^−1^, Sanofi Santé, France).

#### Inflammatory cytokines and markers of oxidative stress in the blood

Immediately after anaesthetisation, blood samples were obtained from the low-risk population by direct intra-aortic puncture for assays of IL-1β, IL-6, thiobarbituric acid reactive substances (TBARs) and glutathione peroxidase. Blood was collected in sterile 4-ml tubes containing lithium heparin (BD Vacutainer®, BD-Plymouth, UK). Within 30 minutes of sample collection, plasma was separated by simple centrifugation at 1200 g and 4 °C for 15 minutes. The supernatant was stored at −80 °C until testing. For the TBARS test, protein was removed from the plasma before storage: 100 µl of plasma was transferred into a 1.5-ml Eppendorf tube, and 200 µl iced 10% TCA was added. After 5 minutes of incubation, the tube was spun at 14.000 rpm for 5 minutes. This supernatant was also stored at −80 °C until testing.

Inflammatory cytokines and markers of oxidative stress were assayed using a Bioplex100 (Biorad Inc., CA, USA) and a series of kits: Rat IL-1β ELISA Kit, Rat IL-6 ELISA Kit (Sigma-Aldrich, MO, USA), QuantiChrom TBARS Assay Kit and EnzyChrom Glutathione Peroxidase Assay Kit (BioAssay Systems, CA, USA). Samples, standards and quality controls were all run in duplicate. All standards and quality controls were prepared as recommended by the supplier.

#### Short-chain fatty acids in the contents of the caecum

Immediately before sacrifice, the caecum was separated from the rest of the digestive tract after ligation. The caecum was weighed and then opened up using a scalpel. Part of its contents was placed in a 1.5- ml Eppendorf tube and frozen at −80 °C. Short-chain fatty acids (acetate, propionate, isobutyrate, butyrate, isovalerate, valerate) were assayed in the frozen sample by gas chromatography–mass spectrometry (GC-MS). The concentration of each short chain fatty acid was expressed as a percentage of the total concentration of short-chain fatty acids in the caecum.

### Statistical analysis

Numerical data were expressed as median [interquartile interval] for quantitative variables and as a percentage [95% confidence interval] for binary variables. A contingency table was used for independence and association tests coupled with an exact Fisher or Chi-squared test. A Mann-Whitney test was used to analyse differences between unpaired groups, and a Wilcoxon test was used for comparisons within paired groups. Spearman’s test was used to investigate correlations. Differences were considered as significant if the p-value was less than 0.05. Statistical calculations were performed using XLSTAT-Pro® software (Addinsoft, Paris, France).

## Electronic supplementary material

Supplementary information
