# Supplementary material for: Stimulating fermentation by the prolonged acceleration of gut transit protects against decompression sickness
Source: Sci Rep. 2018 Jul 4;8:10128. doi: 10.1038/s41598-018-28510-x (PMC6031626; doi:10.1038/s41598-018-28510-x)
Supplement: Supplementary file 1 — Supplementary information [file 41598_2018_28510_MOESM1_ESM.pdf]

STIMULATING FERMENTATION BY THE PROLONGED ACCELERATION OF GUT TRANSIT PROTECTS AGAINST DECOMPRESSION SICKNESS

Authors : de MAISTRE[1*] Sébastien, VALLEE[2] Nicolas, GAILLARD[3] Sandrine, DUCHAMP[4] Claude, BLATTEAU[1] Jean-Eric.

Affiliation :

[1] Service de Médecine Hyperbare et Expertise Plongée, HIA Sainte-Anne, BP600, Toulon Cedex 9, France

[2] Équipe Résidente de Recherche Subaquatique Opérationnelle, Institut de Recherche Biomédicale des Armées, BP 600, Toulon Cedex 9, France

[3] Biotech Services, Université de Toulon, CS 60584, Toulon Cedex 9, France

[4] LEHNA, UMR 5023-CNRS/UCBL, Université Claude Bernard Lyon 1, 43 Bd du 11 Novembre 1918, Villeurbanne Cedex, France

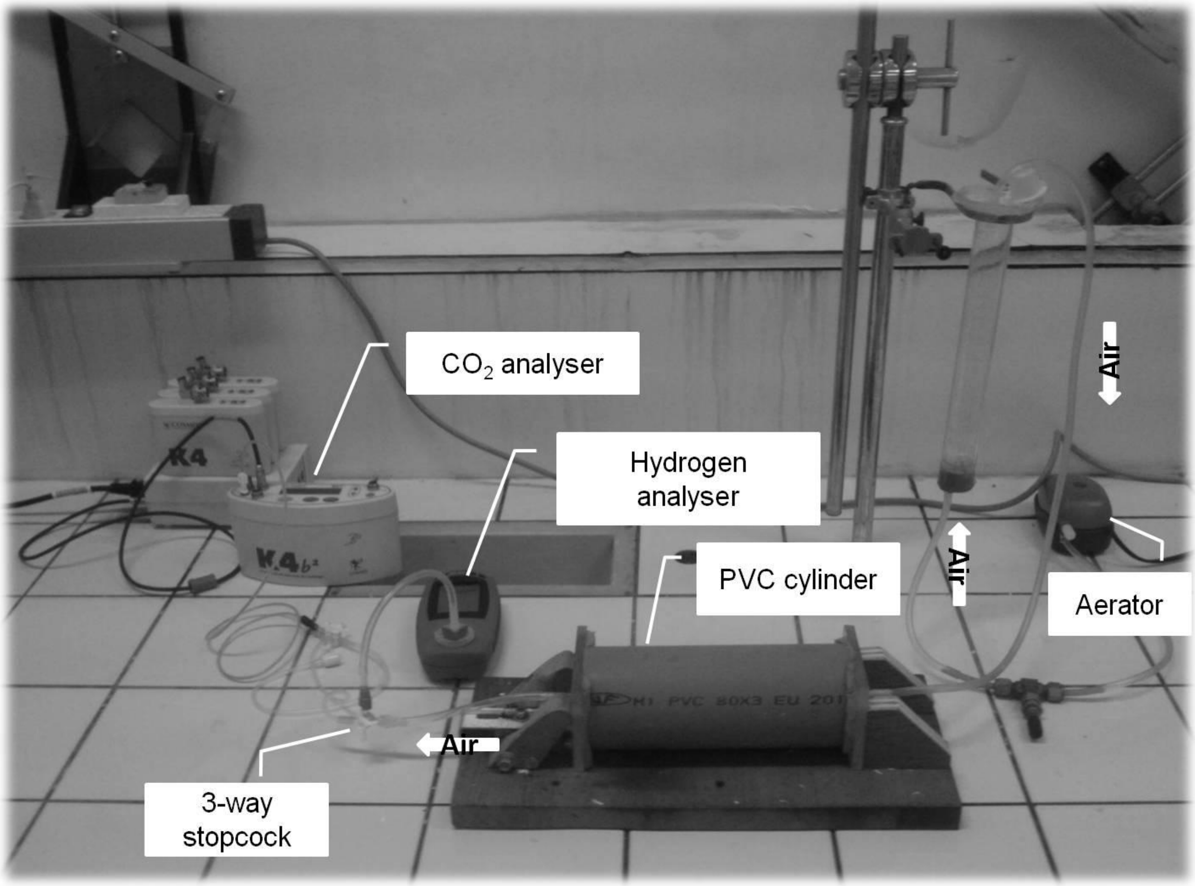

Figure: Installation used for the measurement of hydrogen and carbon dioxide in exhaled air in rats.
